# Usefulness of modified S-line for upper instrumented vertebra selection in adolescent idiopathic scoliosis Lenke type 2 curves

**DOI:** 10.1038/s41598-022-21274-5

**Published:** 2022-10-10

**Authors:** Tetsuhiko Mimura, Shota Ikegami, Tomohiro Banno, Shoji Seki, Tetsuro Ohba, Hiroki Oba, Shugo Kuraishi, Masashi Uehara, Ryo Munakata, Takashi Takizawa, Terue Hatakenaka, Takayuki Kamanaka, Yoshinari Miyaoka, Daisuke Kurogochi, Takuma Fukuzawa, Hirotaka Haro, Yoshiharu Kawaguchi, Yukihiro Matsuyama, Michihiko Koseki, Jun Takahashi

**Affiliations:** 1 Department of Orthopaedic Surgery, Shinshu University School of Medicine, Asahi 3-1-1, Matsumoto, Nagano 390-8621 Japan; 2 Department of Orthopaedic Surgery, Hamamatsu University School of Medicine, Shizuoka, Japan; 3 Department of Orthopaedic Surgery, Faculty of Medicine, University of Toyama, Toyama, Japan; 4 Department of Orthopaedic Surgery, University of Yamanashi School of Medicine, Yamanashi, Japan; 5 Faculty of Textile Science and Technology, Shinshu University, Nagano, Japan

**Keywords:** Medical research, Paediatric research, Paediatrics, Therapeutics, Orthopaedics

## Abstract

No validated systems exist for selecting the upper instrumented vertebra (UIV) for optimal postoperative shoulder balance in Lenke type 2 adolescent idiopathic scoliosis (AIS). This study evaluated a new method for shoulder balance prediction using the modified Shinshu line (MSL) for UIV selection in AIS Lenke type 2 curves. Fifty-five consecutive AIS patients receiving posterior spinal fusion (PSF) for a Lenke type 2 AIS curve were retrospectively analyzed according to several UIV determination models. Shoulder imbalance was judged as absolute radiographic shoulder height ≥ 10 mm at the 2-year observational endpoint. The MSL was the line between the center of the spinous process of C7 and that of the lowest instrumented vertebra. The vertebral body first touched proximally by the MSL was defined as the MSL vertebra (MSLV) and recommended as the UIV. The group with the UIV matching the MSLV had a significantly lower prevalence of shoulder imbalance of 23% (odds ratio 4.08, 95% CI 1.22–13.7, *P* = 0.02). Setting the MSLV as the UIV in PSF for AIS Lenke type 2 may reduce the prevalence of postoperative shoulder imbalance.

## Introduction

Adolescent idiopathic scoliosis (AIS) is a three-dimensional deformity of the spine that affects the rib cage, pelvis, and shoulder girdle. Shoulder height imbalance is a significant potential complication after posterior spinal fusion (PSF) in patients with Lenke type 1 or 2 AIS, resulting in diminished cosmetic results, patient dissatisfaction, and in some cases, reoperation^1^. One of the main determinants of post-surgical shoulder balance is the selection of the upper instrumented vertebra (UIV)^2,3^. However, few validated systems exist on selecting the UIV for optimal postoperative shoulder balance, especially in Lenke type 2 AIS.

Trobisch et al. recommended fusion to T2 for all Lenke type 2 curves^4^. However, T2 selection as the UIV does not guarantee postoperative shoulder balance at the two-year follow-up^5,6^. Routine fusion to T1 or T2 is also less desirable due to concerns of operative time, blood loss, upper extensor muscle dissection and denervation, and scar visibility on the lower neck^7^. Several other methods have been proposed for UIV selection in Lenke type 2 curves^8,9^. In their review, Bjerke et al. could not identify a specific set of UIV selection criteria that accurately predicted post-surgical shoulder balance, and commented that additional validated measures were needed^10^.

The modified Shinshu line (MSL) is defined as the line between the center of the spinous process of C7 and that of the lowest instrumented vertebra (LIV), with the vertebral body first touching the MSL proximally determined as the MSL vertebra (MSLV) and recommended as the UIV^11^. The concept of the MSLV model is to set the UIV such that the LIV and C7 are in a straight line, with the aim of the central sacral vertical line (CSVL) and C7 plumb line (C7PL) coinciding after corrective PSF while minimizing the fused area. In the PSF for Lenke type 1A AIS, not only better trunk balance, but also improved shoulder balance, were obtained without a reduction in correction rate by setting the MSLV as the UIV^11^. We hypothesize that this concept can be applied to Lenke type 2 AIS as well. Therefore, this study aimed to clarify if the MSLV method was suitable for UIV selection prior to Lenke type 2 AIS surgery in terms of predicting shoulder balance.

## Patients and methods

This study was approved by the institutional ethics review board of Shinshu University Hospital (No. 5097) and conducted in accordance with the ethical standards of the Declaration of Helsinki.

### Patient population

We retrospectively reviewed the medical records of all AIS patients who underwent PSF at among four university hospitals between December 2006 and March 2018. The inclusion criteria were as follows: (1) diagnosis of AIS with a type 2 curve according to the Lenke classification system^2^, and (2) receiving one-stage PSF using the rod rotation and direct vertebral rotation technique with an all-pedicle screw construct. The exclusion criteria were: (1) UIV above T2, and (2) follow-up period of less than 2 years. Informed consent was obtained from all patients and guardians.

### Outcome measures

**Figure 1:**
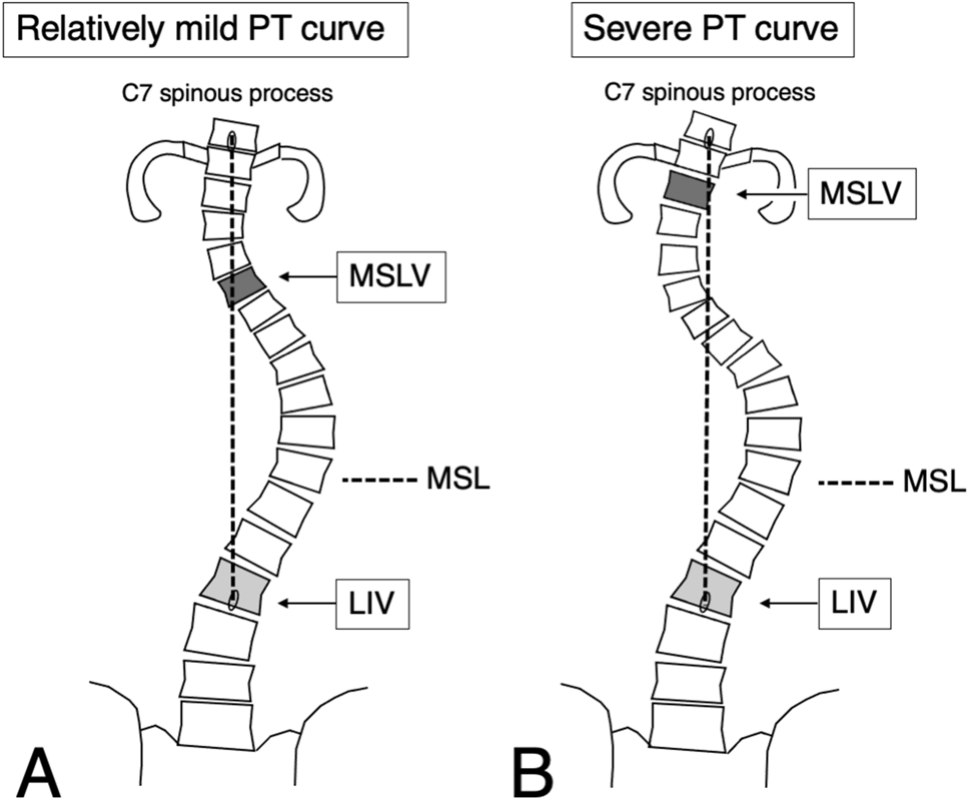
The MSL is the line connecting the center of the spinous process of C7 with that of the LIV. The vertebral body first touching the MSL proximally is defined as the MSLV. (**A**) A relatively mild PT curve. (**B**) For severe PT curves, the line may deviate from the vertebral column, in which case we redefined the MSLV as the most proximal vertebral body first touching the MSL. *PT* proximal thoracic, *LIV* lowest instrumented vertebra, *UIV* upper instrumented vertebra, *MSL* modified Shinshu line, *MSLV* MSL vertebra.

Standing long-cassette postero-anterior (PA) and lateral radiographs taken before surgery and at 2 years postoperatively were evaluated by a trained orthopedic surgeon who was not involved in the surgeries. Three-dimensional evaluations were not performed in this study. The MSL was originally defined as the line between the center of the spinous process of C7 and that of the LIV (Fig. 1A)^11^. The vertebral body first touched proximally by the MSL was defined as the MSLV. For severe proximal thoracic (PT) curves, the MSL might deviate from the vertebral column, in which case we redefined the MSLV as the most proximal vertebral body first touching the MSL (Fig. 1B). The MSLV was determined using preoperative standing PA whole-spine radiographs immediately before surgery. In this method, the MSLV was the recommended UIV for surgery. The group in which the UIV matched the MSLV was defined as the matched group (M-group), the group in which the UIV was proximal to the MSLV was defined as the proximal group (P-group), and the group in which the UIV was distal to the MSLV was classified as the distal group (D-group). As the D-group contained only two patients, statistical analyses including this group were considered inappropriate. Radiographic parameters and Scoliosis Research Society (SRS)-22r findings were compared between the M-group and the P-group.

We evaluated Cobb angle of the PT and main thoracic (MT) curves, correction rate, absolute length between the C7PL and CSVL (C7PL-CSVL), clavicular angle (CA), T1 tilt, absolute radiographic shoulder height (|RSH|), MT apical vertebral translation, T5-12 thoracic kyphosis, T12-S1 lumbar lordosis, proximal junction kyphosis (PJK), distal adding-on, and SRS-22r questionnaire results preoperatively and 2 years postoperatively. PJK was diagnosed as the proximal junction sagittal Cobb angles between the lower endplate of the UIV and the upper endplates of the two supradjacent vertebrae being: (1) more than 10°, and (2) at least 10° greater than preoperative measurements at 2 years postoperatively^12^. Distal adding-on was defined as: (1) an increase in Cobb angle ≥ 5° and distalization of the end vertebra, or (2) a change in disc angulation ≥ 5° below the LIV between initial and 2-year follow-up radiographs^13^.

Shoulder imbalance was defined as |RSH|≥ 10 mm at 2-year follow-up^14–16^. Based on this definition, the cohort was divided into the balanced group and imbalanced group for comparisons of radiographic parameters and SRS-22r findings.

Using the MSLV method, a recommended UIV was determined for each patient. The UIV recommendations of the MSLV and other methods were then compared to the actual UIV instrumented. A matched selection was in agreement with the method’s recommendation, and an unmatched selection was one that differed. We analyzed three other modes of selecting the UIV with regard to shoulder balance according to previous reports: Lenke^8^, Ilharreborde^9^, and Trobisch^4^. In the Lenke method, fusion to T2 is recommended for left shoulder elevation. Ilharreborde et al. described a system for evaluating Lenke 1 and 2 curves based on preoperative PT and MT Cobb angles on bending radiographs, T1 tilt, and RSH, with a table for inclusion of the curve to T1, partial inclusion to T2 or T3, or no inclusion (T4 or below) (Table 1). The Ilharreborde method’s recommendation of fusion to T1 was considered to be correct if the actual fusion was to T2 in the same manner as in a prior report^10^. Lastly, Trobisch et al. recommended fusion to T2 for all Lenke type 2 curves.

**Table 1:** Ilharreborde recommendation modified by Bjerke et al.

	T1 tilt and RSH to the left	T1 tilt and shoulder balance in opposite direction	T1 tilt and RSH to the right
α-(β/2) < 15°	T4 or below	T2 or T3	T1 or T2
α-(β/2) > 15°	T2 or T3	T1 or T2	T1 or T2

α is the Cobb angle of the proximal thoracic curve on lateral bending towards the convex side (left side). β is the Cobb angle of the main thoracic curve on lateral bending towards the convex side (right side).

*RSH* radiographic shoulder height.

### Statistical analysis

Comparisons of mean values between two groups were performed using Welch's *t*-test. Statistical analysis for categorical variables (e.g., proportions of matched and unmatched shoulder outcomes and gender) was performed by Fisher's exact test. Ordinal data (e.g., UIV and MSLV) were analyzed using the Mann–Whitney U test. Considering the confounding variable of preoperative CA on shoulder imbalance, multivariate logistic regression analysis was employed, adopting groups based on each method and preoperative CA as an explanatory variable. The kappa coefficient was calculated to estimate interobserver reliability for the MSLV method. For all analyses, a *P* value < 0.05 was considered significant. Statistical testing was performed using the statistical package R, version 3.6.1 (available at http://www.r-project.org). The Mann–Whitney U test was conducted using R package exactRankTests, version 0.8-35. The figure was generated using Microsoft PowerPoint for Mac, version 16.64.

## Results

Fifty-eight consecutive AIS patients who underwent PSF for a Lenke type 2 curve were included. All patients completed the 2-year observation period. Three cases were excluded: two in which the UIV was T1 and one where the UIV was C7. Ultimately, 55 patients (45 female and 10 male) were retrospectively investigated. Mean ± standard deviation age was 14.3 ± 1.9 years (range 11–20 years). Lenke type was 2A in 39 patients, 2B in 10 patients, and 2C in six patients. For Lenke 2A, the LIV was the vertebra that last touched the CSVL. For Lenke 2B and 2C curves, the LIV was selected according to previous literature^17^. Briefly, when the stable vertebra (SV) was below the end vertebra (EV), the LIV was chosen as the SV. In the opposite scenario, the LIV was chosen as the EV. If the SV and EV were the same, the LIV was selected as one level below the SV/EV. The LIV was T11 in one patient, T12 in four patients, L1 in 22 patients, L2 in 24 patients, L3 in three patients, and L4 in one patient. UIV selection was based on the preference of each surgeon and institution and was T2 in 43 patients, T3 in four patients, T4 in six patients, T5 in one patient, and T6 in one patient. No PJK or distal adding-on was observed in any patient.

The kappa coefficient for the MSLV method was 0.77, indicating good agreement. The MSL deviated from the vertebral column in 19/55 (34.5%) cases (Fig. 1B), among which 18 had MSLV = T2 and one had MSLV = T3. Among the 55 consecutively treated patients, the M-group contained 22 cases, the P-group contained 31 cases, and two cases were classified into the D-group. One, three, and one patient in the M-, P-, and D-group, respectively, did not complete the SRS-22r questionnaire and were excluded from the analysis of clinical scores. The patient characteristics of the three groups are summarized in Table 2. Preoperatively, the M-group had significantly higher PT Cobb angle and T1 tilt than did the P-group. There were no significant differences in postoperative PT or MT correction rates, C7PL-CSVL, or sagittal alignment between the groups. Postoperatively, although CA and T1 tilt did not differ remarkably, the M-group had a significantly lower increase in CA and higher improvement in T1 tilt versus the P-group. We also noted the tendency for an improvement in mean |RSH| in the M-group. In regard to clinical scores, although not significant, improvement of self-image was higher in the M-group than in the D-group.

**Table 2:** Patient characteristics, radiographic data, and SRS-22r scores among the test groups.

	M-group			P-group			D-group			*P* value*
	n	Mean	SD	n	Mean	SD	n	Mean	SD	
Age (years)	22	14.4	2.0	31	14.0	1.9	2	13.0	0.0	0.96
**Sex**										0.30
Female	16			27			2			
Male	6			4			0			
**UIV**										0.18
T2	16			27			0			
T3	1			2			1			
T4	4			2			0			
T5	1			0			0			
T6	0			0			1			
**MSLV**										< 0.01
T2	16			0			2			
T3	1			3			0			
T4	4			4			0			
T5	1			14			0			
T6	0			10			0			
**Preoperative radiographic data**										
PT Cobb angle (°)	22	46.0	11.6	31	38.8	8.0	2	45.5	4.9	0.01
MT Cobb angle (°)	22	55.6	11.9	31	55.5	8.5	2	62.0	7.1	0.95
C7PL-CSVL (cm)	22	0.5	2.0	31	0.7	1.5	2	0.6	0.2	0.72
CA (°)	22	0.6	3.3	31	− 0.3	2.3	2	5.0	4.2	0.25
T1 tilt (°)	22	9.7	8.8	31	5.0	6.4	2	13.0	1.4	0.04
|RSH| (mm)	22	10.4	6.4	31	8.9	6.9	2	22.0	4.2	0.43
MT AVT (cm)	22	4.4	1.5	31	5.0	1.6	2	5.9	0.3	0.22
T5-12 thoracic kyphosis	22	12.5	8.3	31	14.2	10.9	2	17.5	0.7	0.53
T12-S1 lumbar lordosis	22	42.9	9.4	31	51.1	8.2	2	40.0	2.8	< 0.01
**2-year postoperative radiographic data**										
PT Cobb angle (°)	22	21.0	11.8	31	18.7	6.3	2	26.5	3.5	0.41
PT correction rate (%)	22	55.1	18.0	31	50.3	17.4	2	41.8	1.4	0.34
MT Cobb angle (°)	22	21.6	9.7	31	17.8	7.1	2	33.0	1.4	0.13
MT correction rate (%)	22	61.1	16.1	31	67.4	13.1	2	46.3	8.4	0.13
C7PL-CSVL (cm)	22	0.1	1.6	31	0.1	1.2	2	0.1	0.1	0.98
CA (°)	22	2.0	2.5	31	3.2	2.1	2	5.5	4.9	0.07
ΔCA (°)	22	1.4	2.7	31	3.5	2.9	2	0.5	0.7	< 0.01
T1 tilt (°)	22	7.0	5.0	31	6.7	4.2	2	6.0	0.0	0.83
ΔT1 tilt (°)	22	− 2.7	7.3	31	1.7	6.0	2	− 7.0	1.4	0.02
|RSH| (mm)	22	8.4	7.9	31	11.6	7.5	2	13.0	7.1	0.14
Δ|RSH| (mm)	22	− 2.0	8.3	31	2.7	9.9	2	− 9.0	2.8	0.07
MT AVT (cm)	22	1.6	1.0	31	1.2	0.8	2	3.1	1.2	0.11
T5-12 thoracic kyphosis	22	19.3	6.1	31	21.9	9.7	2	20.0	8.5	0.24
T12-S1 lumbar lordosis	22	44.3	9.1	31	46.5	10.0	2	42.0	0.0	0.41
**Preoperative SRS-22r scores**										
Pain	21	4.2	0.7	28	4.2	0.7	1	3.2	NA	0.90
Function	21	4.3	0.5	28	4.5	0.5	1	4.6	NA	0.36
Self-image	21	2.7	0.5	28	3.0	0.6	1	2.6	NA	0.01
Mental health	21	4.1	0.7	28	3.8	0.8	1	4.6	NA	0.34
Subtotal	21	3.9	0.5	28	3.9	0.5	1	4.2	NA	0.95
**2-year postoperative SRS-22r scores**										
Pain	21	4.5	1.0	28	4.3	0.7	1	4.8	NA	0.33
Function	21	4.7	0.3	28	4.7	0.3	1	4.2	NA	0.91
Self-image	21	4.1	0.6	28	4.1	0.6	1	3.2	NA	0.94
Mental health	21	4.4	0.6	28	4.3	0.6	1	5.0	NA	0.46
Subtotal	21	4.4	0.4	28	4.4	0.4	1	4.3	NA	0.51
Satisfaction	21	4.4	0.6	28	4.1	0.8	1	3.0	NA	0.14
**Change in SRS-22r scores**										
Pain	21	+ 0.4	0.6	28	+ 0.1	0.8	1	+ 1.6	NA	0.23
Function	21	+ 0.4	0.4	28	+ 0.2	0.6	1	− 0.4	NA	0.22
Self-image	21	+ 1.4	0.8	28	+ 1.1	0.7	1	+ 0.6	NA	0.09
Mental health	21	+ 0.4	0.5	28	+ 0.5	0.9	1	+ 0.4	NA	0.96
Subtotal	21	+ 0.6	0.4	28	+ 0.4	0.6	1	+ 0.1	NA	0.35

*M-group versus P-group.

*UIV* upper instrumented vertebra, *MSLV* modified Shinshu line vertebra, *PT* proximal thoracic, *MT* main thoracic, *C7PL-CSVL* absolute length between C7 plumb line and central sacral vertical line, *CA* clavicular angle, *|RSH|* absolute radiographic shoulder height, *AVT* apical vertebral translation, *SRS-22r* Scoliosis Research Society 22r questionnaire, *NA* not available.

The patient characteristics of the balanced and imbalanced shoulder groups are shown in Table 3. No significant differences were seen for the UIV or MSLV, although the balanced group had a significantly higher proportion of UIV-MSLV matching as compared with the imbalanced group. The imbalanced group had significantly higher preoperative CA. Postoperatively, the balanced group exhibited significantly greater MT Cobb angle, but a similar MT correction rate.

**Table 3:** Patient characteristics, radiographic data, and SRS-22r scores of the balanced group and imbalanced group.

	Balanced group			Imbalanced group			*P* value
	n	Mean	SD	n	Mean	SD	
Age (years)	32	14.4	2.0	23	14.0	1.9	0.52
**Sex**							0.03
Female	23			22			
Male	9			1			
**UIV**							0.24
T2	23			20			
T3	3			1			
T4	5			1			
T5	1			0			
T6	0			1			
**MSLV**							0.12
T2	12			6			
T3	2			2			
T4	7			1			
T5	7			8			
T6	4			6			
**Relationship between UIV and MSLV**							0.02
Matched	17			5			
Unmatched	15			18			
**Preoperative radiographic data**							
PT Cobb angle (°)	32	42.5	11.2	23	41.1	8.4	0.58
MT Cobb angle (°)	32	57.8	9.3	23	52.9	10.2	0.07
C7PL-CSVL (cm)	32	0.7	1.5	23	0.5	1.9	0.64
CA (°)	32	− 0.7	2.8	23	1.5	2.7	< 0.01
T1 tilt (°)	32	5.8	9.0	23	9.0	4.9	0.09
|RSH| (mm)	32	9.7	7.2	23	10.3	6.9	0.73
MT AVT (cm)	32	4.9	1.7	23	4.7	1.4	0.71
T5-12 thoracic kyphosis	32	13.2	8.6	23	14.2	11.2	0.71
T12-S1 lumbar lordosis	32	46.5	8.4	23	48.7	10.9	0.40
**2-Year postoperative radiographic data**							
PT Cobb angle (°)	32	20.7	10.2	23	18.8	6.9	0.42
PT correction rate (%)	32	51.0	18.0	23	53.2	17.1	0.64
MT Cobb angle (°)	32	22.2	9.1	23	16.7	7.1	0.01
MT correction rate (%)	32	61.7	14.8	23	67.4	14.5	0.15
C7PL-CSVL (cm)	32	0.0	1.2	23	0.3	1.5	0.39
CA (°)	32	1.5	1.8	23	4.6	2.1	< 0.01
ΔCA (°)	32	2.2	3.0	23	3.0	2.9	0.29
T1 tilt (°)	32	6.4	4.6	23	7.2	4.2	0.51
ΔT1 tilt (°)	32	0.6	7.4	23	− 1.8	5.8	0.17
|RSH| (mm)	32	5.1	3.2	23	17.7	5.9	< 0.01
Δ|RSH| (mm)	32	− 4.6	6.9	23	7.3	8.1	< 0.01
MT AVT (cm)	32	1.6	1.0	23	1.2	0.9	0.10
T5-12 thoracic kyphosis	32	21.0	8.1	23	20.5	8.9	0.83
T12-S1 lumbar lordosis	32	45.3	8.1	23	45.7	11.3	0.89
**Preoperative SRS-22r scores**							
Pain	29	4.1	0.7	21	4.3	0.7	0.25
Function	29	4.4	0.5	21	4.5	0.5	0.41
Self-image	29	2.7	0.6	21	3.1	0.4	0.02
Mental health	29	3.8	0.8	21	4.1	0.7	0.21
Subtotal	29	3.8	0.5	21	4.0	0.4	0.11
**2-year postoperative SRS-22r scores**							
Pain	29	4.4	0.6	21	4.4	0.7	0.88
Function	29	4.7	0.3	21	4.7	0.4	0.72
Self-image	29	4.1	0.6	21	4.2	0.6	0.70
Mental health	29	4.4	0.6	21	4.4	0.7	0.88
Subtotal	29	4.4	0.3	21	4.4	0.5	0.83
Satisfaction	29	4.2	0.8	21	4.2	0.6	0.80
**Change in SRS-22r scores**							
Pain	29	+ 0.3	0.6	21	+ 0.1	0.9	0.24
Function	29	+ 0.3	0.5	21	+ 0.2	0.7	0.50
Self-image	29	+ 1.3	0.7	21	+ 1.0	0.7	0.11
Mental health	29	+ 0.6	0.7	21	+ 0.3	0.8	0.23
Subtotal	29	+ 0.6	0.4	21	+ 0.3	0.6	0.18

*UIV* upper instrumented vertebra, *MSLV* modified Shinshu line vertebra, *PT* proximal thoracic, *MT* main thoracic, *C7PL-CSVL* absolute length between C7 plumb line and central sacral vertical line, *CA* clavicular angle, *|RSH|* absolute radiographic shoulder height, *AVT* apical vertebral translation, *SRS-22r* Scoliosis Research Society 22r questionnaire.

The group matching the MSLV method had a significantly lower prevalence of shoulder imbalance of 23% (odds ratio [OR] 4.08, 95% confidence interval [CI] 1.22–13.7, *P* = 0.02) (Table 4). The ORs of the other reported methods were all less than 1.00. Multiple logistic regression analysis to assess for predictors of shoulder imbalance after adjusting for preoperative CA revealed matching with MSLV selection as a significant factor (OR 9.60, 95% CI 1.91–48.3, *P* < 0.01), in contrast to matching with the Lenke method (OR 0.88, 95% CI 0.22–3.49, *P* = 0.85), Ilharreborde method (OR 0.10, 95% CI 0.01–1.00, *P* = 0.05), or Trobisch method (OR 0.56, 95% CI 0.11–2.83, *P* = 0.48).

**Table 4:** Incidence of shoulder imbalance by UIV selection method.

	Imbalanced	Balanced	Odds ratio	95% CI	*P* value
**MSLV method**					
Unmatched	18 (55%)	15 (45%)	4.08	1.22–13.7	0.02
Matched	5 (23%)	17 (77%)			
**Lenke method**					
Unmatched	6 (30%)	14 (70%)	0.45	0.14–1.45	0.26
Matched	17 (49%)	18 (51%)			
**Ilharreborde method**					
Unmatched	1 (8%)	12 (92%)	0.08	0.01–0.64	< 0.01
Matched	22 (52%)	20 (48%)			
**Trobisch method**					
Unmatched	3 (25%)	9 (75%)	0.38	0.09–1.61	0.32
Matched	20 (47%)	23 (53%)			

*MSLV* modified Shinshu line vertebra, *CI* confidence interval.

We also performed a post-hoc analysis to determine whether a difference in LIV selection affected the MSLV decision. The MSLV changed in 13/55 (23.6%) cases when the LIV was hypothetically moved one level proximal and in 5/55 (9.1%) cases when the LIV was hypothetically move one level distal.

## Discussion

This study revealed two important points. First, the M-group displayed a significantly lower increase in CA and higher improvement in T1 tilt than did the P-group, with no remarkable differences in MT or PT correction rate or C7PL-CSVL. Second, the group matching the MSLV method for UIV selection had a significantly lower prevalence of shoulder imbalance at the 2-year observation endpoint. The MSLV method may therefore be able to predict postoperative shoulder balance more reliably than other reported criteria.

Bjerke et al. examined several typical recommendation systems for UIV but could not identify a suitable set of criteria that predicted postoperative shoulder balance^10^. Thus, the MSLV may be the first UIV selector to accurately predict shoulder balance in Lenke type 2 curves. Preoperative CA has been reported as the best radiographic predictor of postoperative shoulder balance^14^. In the present study, matching the UIV with the MSLV was a significant predictor even after adjusting for CA. Although Cao et al. described postoperative distal adding-on to be associated with postoperative shoulder balance^18^, this phenomenon was absent in the current study. We therefore surmised that the above factors did not invalidate the main findings of our investigation.

There are three possibilities as to why the MSLV method displayed a significantly lower prevalence of shoulder imbalance. First, post-surgical spontaneous PT curve correction is associated with postoperative shoulder balance. In Lenke type 1 AIS, it is believed that if UIV is a more cranial side, the fusion area in the non-structural curve becomes longer, and the spontaneous correction of postoperative shoulder balance and PT curve is lost^3^. Spontaneous PT curve correction also consistently occurs after instrumented correction of the MT curve in Lenke type 2 AIS, although to a lesser extent than in Lenke type 1 since spontaneous correction occurs in association with PT curve flexibility^7^. In the MSLV method, when the PT curve was steep, the MSL tended to deviate from the vertebral column in the PT curve, and so the MSLV was T2. In such cases, T2 fixation is more suitable because of diminished flexibility and less likely spontaneous correction. Second, the overcorrection of MT Cobb angle is associated with postoperative shoulder balance. In Lenke type 1 and 2 AIS curves, it is believed that MT curve overcorrection can result in residual shoulder imbalance^7,19,20^. In the current study, while there was no significant difference in MT correction rate between the M-group and P-group, the M-group group tended to have a lower mean correction rate (61.1% vs. 67.4%). These patients might have had less correction of MT Cobb angle, which could have resulted in better shoulder balance. Third, the continuity of the Lenke type 1 and 2 classifications may have influenced our results. Lenke type 1 and 2 are distinguished by bending PT Cobb angle. It was reported that since conventional supine side-bending radiographs did not reflect the true flexibility of the PT segment^21^, the difference between Lenke type 1 and 2 may be ambiguous. Therefore, it might be preferable to consider that there are cases either similar to Lenke type 1 (Fig. 1A) or better fixed more proximally (Fig. 1B). The MSLV, which has already been found as useful for Lenke type 1^11^, shows promise as a reliable predictor of shoulder balance.

Although several methods have used preoperative shoulder balance to determine the UIV, the MSLV model does not directly employ this parameter. Even in the healthy adolescent population, 28% have poor shoulder balance, suggesting that some cases are not caused by a deformity, but rather by the patient's own habits^22^. Accordingly, it may be inappropriate to set the UIV based on preoperative shoulder balance alone. The MSLV is a simple and practical method that reflects multiple factors related to postoperative shoulder balance, including severity of the PT curve and degree of correction, and appears useful in clinical practice.

This study had several limitations. First, it included a patient group of limited size and had a retrospective design. Additional large prospective studies are needed to confirm our results. Second, the selection of fusion area, surgical construct, and surgical technique for deformity correction were not controlled and varied among the participating institutions, which might have confounded our results. Third, differences in LIV selection methodology may influence the decision of the MSLV. Although the method of LIV determination was consistently applied in this study, inconsistent or arbitrary LIV choice may impact MSLV identification. The findings of this report require validation in patient groups including different LIV determination methods. Fourth, there were very few cases of UIV selection distal to the MSLV; thus, the results of such fusion levels remain unclear. In addition, as there was only one case of T5 as the UIV and no cases of T6 as the UIV in the M-group, the outcomes of the UIV set as the MSLV at T5 or T6 are uncertain. Similarly, although the cases in which the MSL deviated from the vertebral column had MSLV = T2, we also encountered one case of MSLV = T3 in this study. It is unclear whether T3 should have been selected in this patient. The accumulation of comparable cases is needed to clarify our results.

In conclusion, the group with matching MSLV and UIV in PSF for Lenke type 2 AIS had a significantly lower prevalence of postoperative shoulder imbalance. The MSLV is a simple and practical approach that may be useful for selecting the UIV in Lenke type 2 AIS patients. Future prospective large-sized studies are needed to validate this UIV selection method for optimal shoulder balance.

## Supplementary Information

Supplementary Information.
